# Correlation between birefringence and absorption mapping in large-size Sapphire substrates for gravitational-wave interferometry

**DOI:** 10.1038/s41598-023-45928-0

**Published:** 2023-12-04

**Authors:** Simon Zeidler, Marc Eisenmann, Marco Bazzan, Pengbo Li, Matteo Leonardi

**Affiliations:** 1 Gravitational Wave Science Project, National Astronomical Observatory of Japan (NAOJ), Tokyo, 181-8588 Japan; 2 Carl Zeiss SMT GmbH, SMT-DCOD, Rudolf-Eber Str. 2, 73447 Oberkochen, Germany; 3 School of Physics and Technology, Wuhan University, Wuhan, 430072 China; 4 Dipartimento di Fisica e Astronomia, Università di Padova, 35131 Padova, Italy; 5 Department of Physics, University di Trento, 38123 Povo, Trento, Italy

**Keywords:** General relativity and gravity, Characterization and analytical techniques, Optical physics

## Abstract

In high-sensitive laser interferometers, such as the gravitational-wave detector KAGRA, ultra-high-quality mirrors are essential. In the case of KAGRA, where cavity mirrors are cooled down to 20 K, large-size Sapphire crystals are used as the substrate for the main mirrors to achieve both a good optical quality (i.e., low absorption and uniform refractive index) and optimized thermal behavior under cryogenic temperatures. To implement the very tight optical specifications required by this demanding application, it is mandatory to test the optical homogeneity of different substrates. In order to characterize refractive-index inhomogeneities of large-size uniaxial samples such as the KAGRA Sapphire test masses, we developed a dedicated setup, allowing to resolve birefringence changes with a sensitivity in the order of $\Delta n\approx 2 \times 10^{-10}$ and a spatial resolution of $1\,{{{\hbox {mm}}}}^{2}$. Moreover, the same setup allows us to simultaneously record residual absorption maps, thus allowing for a comparison between birefringence and absorption features. In this paper, we will present for the first time measurements on a KAGRA-sized Sapphire substrate which has been characterized in terms of absorption already in an earlier work. Both birefringence inhomogeneities and absorption distributions will be compared and correlations discussed.

## Introduction

Sapphire has become an important material in many optical applications. Not only in industry but also in optical science, Sapphire is essential in several delicate instruments and experiments due to its robustness and versatility. In KAGRA^1,2^, the Japanese second-generation gravitational-wave detector, Sapphire plays a key role as well. In contrast to the LIGO detectors^3^ in the USA and the Virgo detector^4^ in Italy, KAGRA uses cryogenic mirrors as test masses inside the laser-interferometer. Under such conditions, Silica (amorphous SiO$_2$), which is the most widely used mirror-substrate material, would not be beneficial as it shows increased internal friction and insufficient thermal conductivity^5^ which would, in any case, harm the detector’s targeted sensitivity due to thermal noise. In order to profit from the cryogenic condition, a material is needed that features good thermal conductivity at low temperatures and has optical as well as mechanical properties fulfilling the specifications required in this kind of application. Sapphire in principle possesses all those characteristics and is therefore one of the materials of election for the next generation of cryogenic gravitational-wave detectors^6^.

Sapphire is a variety of crystalline Al$_2$O$_3$ which crystallizes in a trigonal crystal system. The oxygen anions are arranged in a (slightly distorted) hexagonal close-packing in which the aluminum cations occupy two-thirds of the octahedral interstices^7,8^. In a hexagonal unit cell, we can differentiate between a distinct axis along the hexagon’s height (c-axis) and 3 axes perpendicular to the c-axis representing the 3 symmetry directions of a hexagon’s barrel. This lattice structure basically leads to a non-isotropic behavior of Sapphire crystals when they interact with light. The refractive index is different for the electric field polarized parallel (extraordinary) or perpendicular (ordinary) to the c-axis, hence leading to birefringence.

Nowadays, the growing mechanism of large Sapphire single-crystals with diameters up to 300 mm are quite sophisticated and samples of decent optical quality can be manufactured. The most common techniques to produce large Sapphire crystals are the Czochralski and the Kyropoulos methods where a single crystal is slowly grown from a melt using a condensation seed^8^. Nevertheless, impurities and imperfections within the lattice structure give rise to absorption centers in Sapphire crystals which (from the standpoint of gravitational-wave detectors) are still a limiting factor when compared to manufactured Silica, especially for large-size samples. Particularly Fe, Ni, and Si but also Cr and Ti are common impurities in alumina^8^ whereby their presence is correlated with vacancies in the lattice. When present, Fe, Ni, and Cr ions can substitute Al cation sites^8,9^ but, due to their larger ionic radius, will distort the local lattice structure and hence may induce stress causing refractive index fluctuations due to the elasto-optical effect. In addition, residual thermal induced stress during the manufacturing process may remain inside the lattice and lead to density inhomogeneities or to the formation of dislocation networks which in turn may affect the refractive index as well without altering absorption necessarily. (Please note that the term “thermal induced stress” refers only to stress remaining from temperature inhomogeneities during manufacturing and not the actual temperature of the sample). For smaller-sized Sapphire crystals, these issues may be not so severe as the manufacturer is able to carve from the as-grown boule those regions which are of the highest quality. However, for larger Sapphire crystals, having sizes significantly close to the size of the boule, this is not possible and can cause issues, especially for applications that require highly homogeneous optical properties, as in the case of gravitational-wave interferometry^10^.

The second-generation LIGO and Virgo detectors use high-quality Silica for their test masses confirmed to be of very high optical isotropy. To the best of our knowledge, birefringence effects have not been considered critical for the functioning of those instruments so far. However, in the case of KAGRA and other detectors which are designed to work with crystalline test masses, such as the next-generation cryogenic gravitational-wave interferometers, the problem can become much more serious. Detailed modeling of the effect of birefringence inhomogeneities on KAGRA is a matter of active research which goes beyond the scope of the present paper. It is thus difficult to set a precise requirement on the desired level of birefringence uniformity at this time. However, as a preparatory stage for this study, it is mandatory to validate a method for birefringence characterization on sapphire and other crystalline materials, providing a spatially resolved technique with a sensitivity as high as possible.

In this paper, we will present the results of our efforts to characterize birefringence inhomogeneities for large Sapphire crystals and correlate them with spatially resolved absorption measurements. In the past, there have been efforts already to investigate birefringence in Sapphire in the context of gravitational-wave detector construction. Most noteworthy hereby are the works by Tokunari et al.^11,12^, where an automated measurement system based on a Soleil-Babinet compensator is presented. Compared to their device, we introduced a very comprehensive polarization control without moving parts where we explicitly did not use a polarizer due to their problematic influence on mode shapes but a combination of polarizing beam-splitters and waveplates. With our device we achieved better accuracy in the measured birefringence and improved spatial resolution by a factor $> 10$. In addition, we were successful in combining the capability to measure absorption and birefringence in one system, which is indeed a key feature of our instrument. In section “Experimental setup” of this paper, the main focus will be to describe the features of our measuring system before we turn to a brief overview on the mathematical background of birefringence measurements and the subsequent conclusions on internal stress from the viewpoint of our system. In section “Results”, we are presenting results from measurements on two Sapphire samples, highlighted by those from a KAGRA-sized ($\text {o}\!\!\!{/}:$ 220 mm; thickness: 150 mm) Sapphire bulk. The obtained birefringence map is compared to linear absorption maps obtained with the same setup previously presented in Marchiò et al.^11^, in order to search for a possible correlation between the birefringence distribution in the sample and the presence of absorption centers. In section “Discussion”, we will discuss our results.

## Experimental setup

### Modified PCI

**Figure 1:**
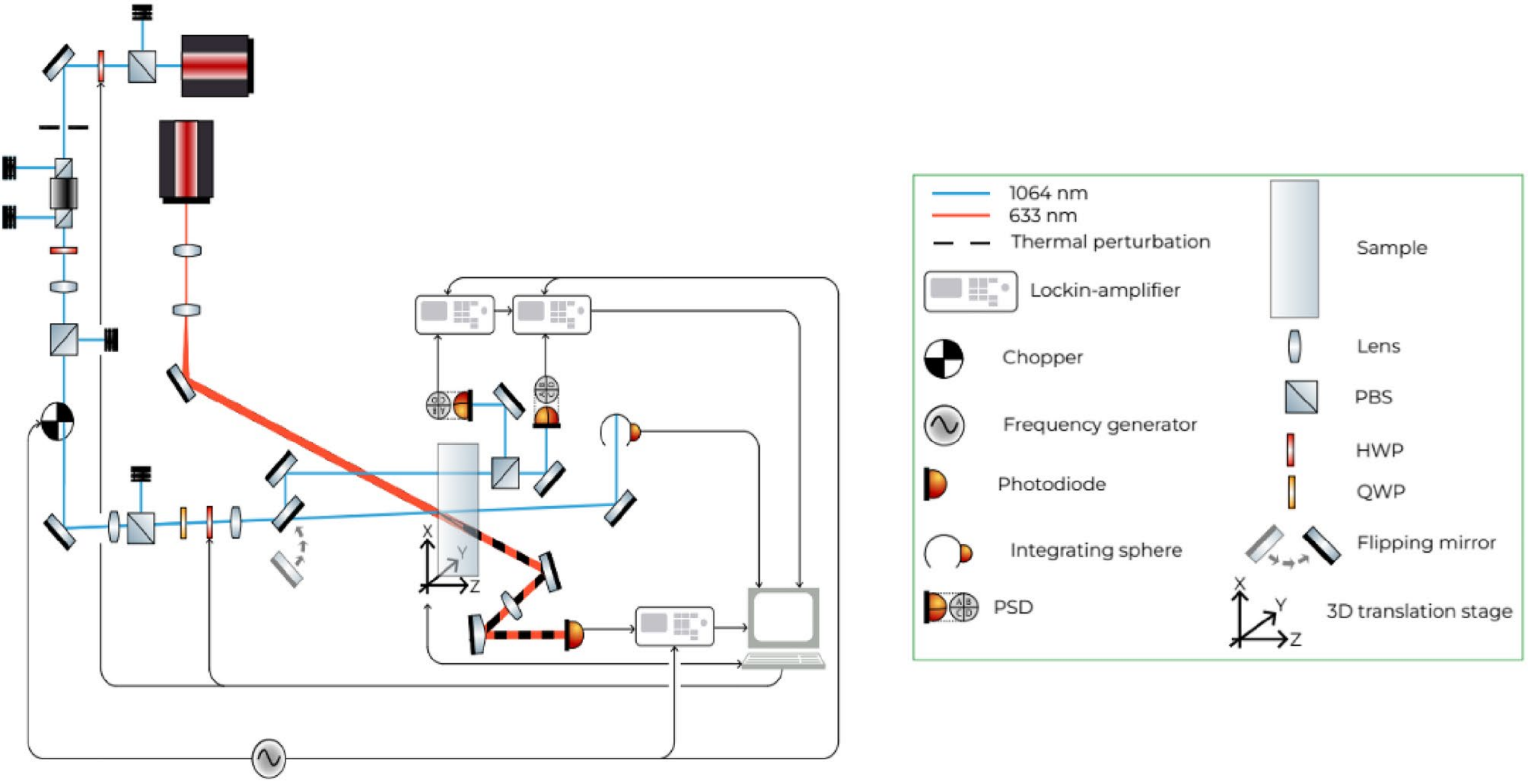
Scheme of both the absorption and birefringence measurement setup. In order to measure birefringence (or absorption), a steering mirror has to be placed (or removed) in front of the sample

At NAOJ, we have established a so-called Photothermal Common-Path Interferometer (PCI)^12^ in order to produce spatially resolved maps of the absorption coefficient for various transparent materials^11^. We designed the system particularly to characterize large mirror substrates that are of concern for KAGRA (cylindrical, 220 mm diameter and 150 mm thickness) but also optics below that size can easily be characterized. We modified the setup slightly to enable measuring birefringence maps by analyzing the polarization state of the exiting beam keeping the possibility to hold and move KAGRA-sized substrates. The pump laser of the PCI (OXIDE LS0100-F1) has stable power when operating at several Watts output (as required for the absorption measurement). However, for birefringence analysis at 1064 nm, this laser has to be used as proof. We added an optical isolation system (Faraday isolator) to protect the laser source from back-reflections and to clean the polarization of the incident beam. Together with a remotely controlled half-wave-plate (HWP) and a polarizing beam-splitter (PBS) in front of the Faraday isolator, we can control the laser power (dynamic range about 1:9000) for both high and low power operation for the absorption and birefringence measurements, respectively. In the following experiments, the power reaching the sample is set at 1.6 mW. In this way, we prevent possible beam distortion due to thermal lensing at high power. We also added a quarter-wave-plate (QWP) and an HWP (remotely controllable) in the beam path which further cleaned the polarization so as to have a linearly polarized beam with a tunable polarization angle (see also Fig. 1 for a detailed sketch showing the setup).

In the original setup of the PCI, the incoming beam possesses an inclination of about $3^\circ$ with respect to the sample’s surface in the Y–Z plane. That inclination is a particular feature of the PCI system and enables absorption measurements without the interference of internal reflections^11^. A non-zero inclination, however, leads to double refraction inside the substrate^8,13^ which complicates considerably the analysis of the results. Therefore, we have put two additional steering mirrors in the input beam path to zero the inclination angle (error: $\le 0.02^\circ$).

The sample is placed on a movable stage that can shift in all three spatial directions (X, Y, and Z, while X is the vertical direction with respect to the laboratory and Z is parallel to the laser beam). Here, we measured both absorption and birefringence every 1 mm in X–Y directions in a circular area of 70 mm radius leading to a measurement duration of about 6h. In order to fix the sample’s position, we have special holders which can be screwed on the stage. For larger samples (from 100 mm diameter), we used structures consisting essentially of two cylindrical bars (oriented in Z direction, 5 to 10 cm in Y apart) in between which the sample is placed. The bars are screwed on two frame plates (front and back) which are fixed on the stage. Hence, the Sapphire substrate is held in place by its own weight whereas silicone-tipped screws on the plates (where the bars are attached) assure further safety. This way, we are certain that external stress can be reduced to a minimum so that our measurements are influenced by internal stress only.

The birefringence measurements are performed by detecting the polarization state of the transmitted beam. We added a new imaging unit close to the one for absorption measurements to detect the S and P-polarized light transmitted through the substrate. This new imaging unit consists of a lens to refocus the beam, a PBS to separate the S and P parts, and after each port of the PBS a steering mirror and a position-sensitive photo-detector. In order to increase the signal-to-noise ratio, we are chopping the laser at about 374 Hz and connecting each photo-detector to a lock-in amplifier to decouple the signal with the chopper frequency. The outputs from the lock-ins as well as the controls are connected to a single PC.

Ideally, the reflection and transmission ports of the PBS correspond to the S and P polarized components of the transmitted beam. However, real PBS have always some small polarization leakages so that $R_s < 1$ and $R_p > 0$ where $R_s$ and $R_p$ are the reflection coefficients of the PBS for the -s and -p components, respectively. Similar inequalities hold also for the transmission coefficients $T_{s(p)}$. Furthermore, the two photo-detectors will have different optical responses which we indicate as $K_s$ and $K_p$ for the photodiodes in the $_s$ and $_p$ output ports respectively. The photodiode’s optical response is sensitive to the position of the beam on the sensor. We are therefore using position-sensitive photodiodes to ensure that the beam is always centered on the sensor. This makes the coefficient $K_{s(p)}$ representative of the intrinsic optical response of the photodiode. The voltage signals $V_s$ and $V_p$ measured by the two photodiodes can therefore be expressed as (neglecting losses):

1 $$\begin{aligned} V_p&= \left( T_p \cdot P_p + T_s \cdot P_s\right) \cdot p(t) \cdot K_p \nonumber \\ V_s&= \left( R_p \cdot P_p + R_s \cdot P_s\right) \cdot p(t) \cdot K_s \end{aligned}$$

where $P_p\cdot p(t)$ and $P_s\cdot p(t)$ are the (integrated) intensities of the -p and s- polarizations of the beam transmitted past the sample, both proportional to the power of the probe beam *p*(*t*). The information on the beam polarization is encoded in the two coefficients $P_p$ and $P_s$, corresponding to the square modulus of the Jones’ vector components for the transmitted beam. To obtain their value we can express Eq. (1) as a matrix equation and invert it to obtain:

2 $$\begin{aligned} p(t)\cdot (P_p, P_s)^t = M^{-1} {(V_p, V_s)}^t \end{aligned}$$

Before starting the birefringence measurement, it is therefore mandatory to measure all the elements of M. This was performed by the following procedure. First, we tuned the input beam’s inclination angle to be normal on the sample and tuned the QWP and HWP rotation angle to minimize $V_p$ with precision smaller than $0.05^\circ$. This corresponds to tuning the input beam to a polarization parallel to X, which we define to be the S polarization (due to the normal impact, S and P are arbitrary and need to be specified). Rotating the controllable HWP by $45^\circ$ will then change the input beam’s polarization from S to P. Measuring $V_p$ and $V_s$ in these two configurations allows for the reconstruction of all the elements of M. Finally, exploiting the fact that $P_s + P_p = 1$, all the data are re-scaled so that their sum is normalized to one. In this way, we can get rid of the power fluctuations of the laser.

Note that the $R_s$ and $R_p$ agree with the manufacturer specifications (Thorlabs, CCM1-PBS25-1064-HP/M). We are able to properly reconstruct $P_s$ and $P_p$ with a relative error of $3 \times 10^{-4}$. As discussed in a later section, this will be the leading source of uncertainty in our birefringence measurement. Following these measurements, it is then possible to measure the birefringence of the sample of interest.

### Birefringence calculation

**Figure 2:**
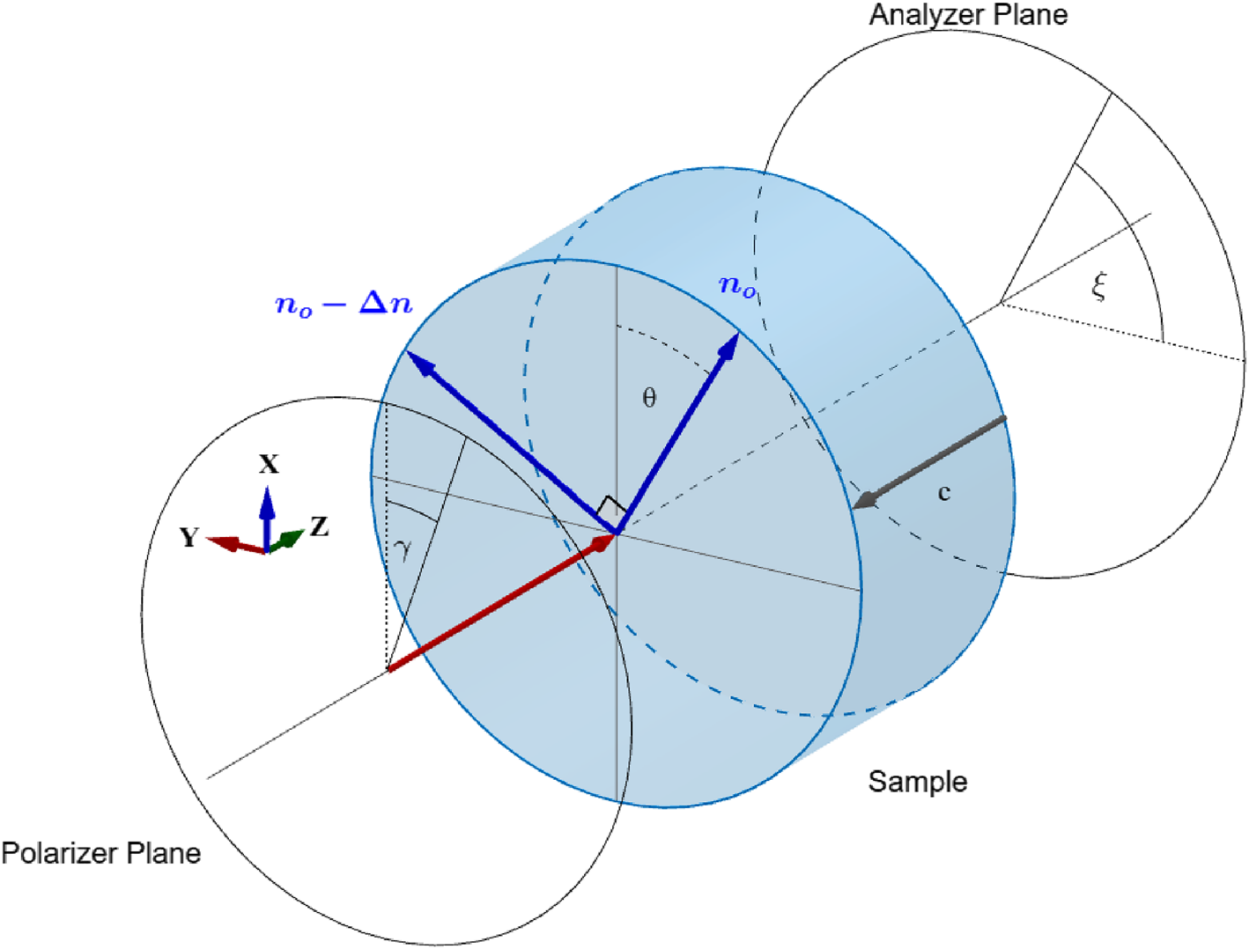
Overview of the denominations and orientations used for the measurements and analysis. The incident beam (red arrow) is linearly polarized having an angle $\gamma$ with respect to the vertical axis (X) in the laboratory and moves along Z from left to right through the sample (the c-axis is oriented parallel to Z). There, internal stress gives rise to a splitting of the refractive index along two axes having an angle $\theta$ with respect to X (or Y, respectively), forming a new coordinate system (blue) for the incoming beam. The beam’s electric field is split into two components, parallel to each of these axes. Due to the difference in the refractive indexes along these axes ($\Delta n$), the beam’s polarization changes which can be measured with the PBS splitting the transmitted beam’s electric field into its S and P polarization.

Sapphire is an uniaxial crystal that behaves optically isotropic for light propagating it along the c-axis which in the case of KAGRA is the ideal case. Any miscut angle between the c-axis and surface or internal misalignment of the c-axis, however, will lead to birefringence. In addition, internal stress can alter the optical indicatrix of the crystal via the elasto-optic effect. Assuming a plane-parallel sample and a beam propagating along a surface normal direction, those effects come down to introduce a position-dependent birefringence. In the following, we will consider that the Sapphire samples are characterized by a spatially modulated birefringence perturbation ($\Delta n$) superimposed to a constant intrinsic birefringence background possibly due to the presence of miscut. We assume that this spatial-dependent birefringence modulation is induced by the presence of stresses. Furthermore, as we are measuring the polarization components after transmission through the entire sample, we are only sensitive to the X–Y plane birefringence while it is integrated along the Z direction (Fig. 2).

Our device is basically a *plane-* or *linear-polariscope*^14,15^: we inject light with a known polarization into a sample and analyze the transmitted polarization relative to it (see also Fig. 2). However, in usual plane polariscopes, one would adjust the input polarizer perpendicular to the output polarizer (analyzer) and rotate the sample^14^. In our case, the incident polarization is rotated and we use a PBS instead of an analyzer. In this section, we will show how information may be obtained with this kind of measurement.

As for the definition of a proper reference frame, we initially assume our probe beam is propagating through the sample in direction Z and the c-axis of the Sapphire sample is parallel to that direction (i.e. no miscut).

Thus, we set the (unperturbed) dielectric tensor $\varepsilon$ in this reference frame as

3 $$\begin{aligned} \varepsilon = \begin{pmatrix} \varepsilon _o &{}\quad 0 &{}\quad 0\\ 0 &{}\quad \varepsilon _o &{}\quad 0\\ 0 &{}\quad 0 &{}\quad \varepsilon _e \end{pmatrix}, \end{aligned}$$

where $\varepsilon _o = 1/n_o$, $\varepsilon _e = 1/n_e$ (see also Fig. 2 for a description of the reference frames and denominations). In this condition, the index-ellipsoid (indicatrix) seen by the input beam has a circular section and the sample is optically isotropic for the beam propagating along the Z direction. Any stress perturbation induces a modification of the sample’s birefringence via the photo-elastic effect. In this case, the indicatrix will be deformed by a quantity $\Delta n$ and tilted by an angle $\theta$ as shown in Fig. 2. Our goal is thus to estimate those two quantities with our measurements.

In order to solve the problem for $\theta$ and $\Delta n$, we need at least two maps with different input polarization angles. Defining the input polarization angle as $\gamma$ (measured as off-angle from the vertical direction in our global coordinate system), we have $\gamma _0$ and $\gamma _1$ for the two maps. Let $P_0$ and $P^0_s$ be the powers of the input beam and the S-polarized output beam for the case $\gamma _0$ (we assume that the areas receiving the respective beams do not change). From a rigorous consideration, it can be shown that:

4 $$\begin{aligned} \sin (\pi /\lambda \cdot d\cdot \Delta n)^2 = \frac{P_s^0 - \cos (\gamma _0)^2}{\cos (\gamma _0)\sin (\gamma _0)\sin (4\theta ) - \sin (2\theta )^2\cos (2\gamma _0)}, \end{aligned}$$

with $\lambda$ the wavelength of the laser. For any other $\gamma _1$, we can in the same way conclude

5 $$\begin{aligned} \begin{aligned} P_s^1 = \cos (\gamma _1)^2 + \frac{P_s^0 - \cos (\gamma _0)^2}{\cos (\gamma _0)\sin (\gamma _0)\sin (4\theta ) - \sin (2\theta )^2\cos (2\gamma _0)} \cdot \bigg [ \cos (\gamma _1)\sin (\gamma _1)\sin (4\theta ) - \sin (2\theta )^2\cos (2\gamma _1)\bigg ], \end{aligned} \end{aligned}$$

where we have used already the outcome of Eq. (4).

The above equation can be rearranged to form an analytic expression for $\theta$:

6 $$ \begin{aligned}    & \frac{1}{2}\tan (2\theta ) = \frac{{\cos (\gamma _{1} )\sin (\gamma _{1} ) - R\cos (\gamma _{0} )\sin (\gamma _{0} )}}{{\cos (2\gamma _{1} ) - R\cos (\gamma _{0} )}}, \\     & {\text{with:}} \\     & R = \frac{{P_{s}^{1}  - \cos (\gamma _{1} )^{2} }}{{P_{s}^{0}  - \cos (\gamma _{0} )^{2} }}. \\  \end{aligned}  $$

Thus, Eqs. (4) and (6) can be used to extract $\theta$ and $\Delta n$ from two measurements. Iwaki and Koizumi^16^ come to similar results in spite of the fact that they were using cubic silicon as sample. This can be understood due to the fact that we are basically measuring the projection of the indicatrix on the XY-plane, where the optical indicatrix in absence of refractive index perturbations is isotropic, similar to cubic crystals. It should be noted that the inversion of Eq. (6) requires that the right-hand side is always comprised between $\pm \, \pi /4$. While this is always true in theory, in practice some pathological cases can appear for unfavorable combinations of $\gamma _0$, $\gamma _1$, and for low signal levels, leading to near-zero values of the denominator. In those cases, the measurement becomes very sensitive to noise and the inversion procedure may fail. Whenever this happens, it is sufficient to repeat the analysis by choosing another combination of $\gamma _0$ and $\gamma _1$. Also, note that $\Delta n$ can only be determined to its absolute value. We cannot decide whether it is positive or negative with our method. Hence, all values of $\Delta n$ being presented here are actually $|\Delta n|$.

## Results

### Sample characterization

The sample presented here is the same one characterized already by the paper from Marchiò et al.^11^. It is a Sapphire single-crystal (cylindrical cut with the c-axis perpendicular to the two main surfaces) with a diameter of 220 mm and a thickness of 150 mm.

**Figure 3:**
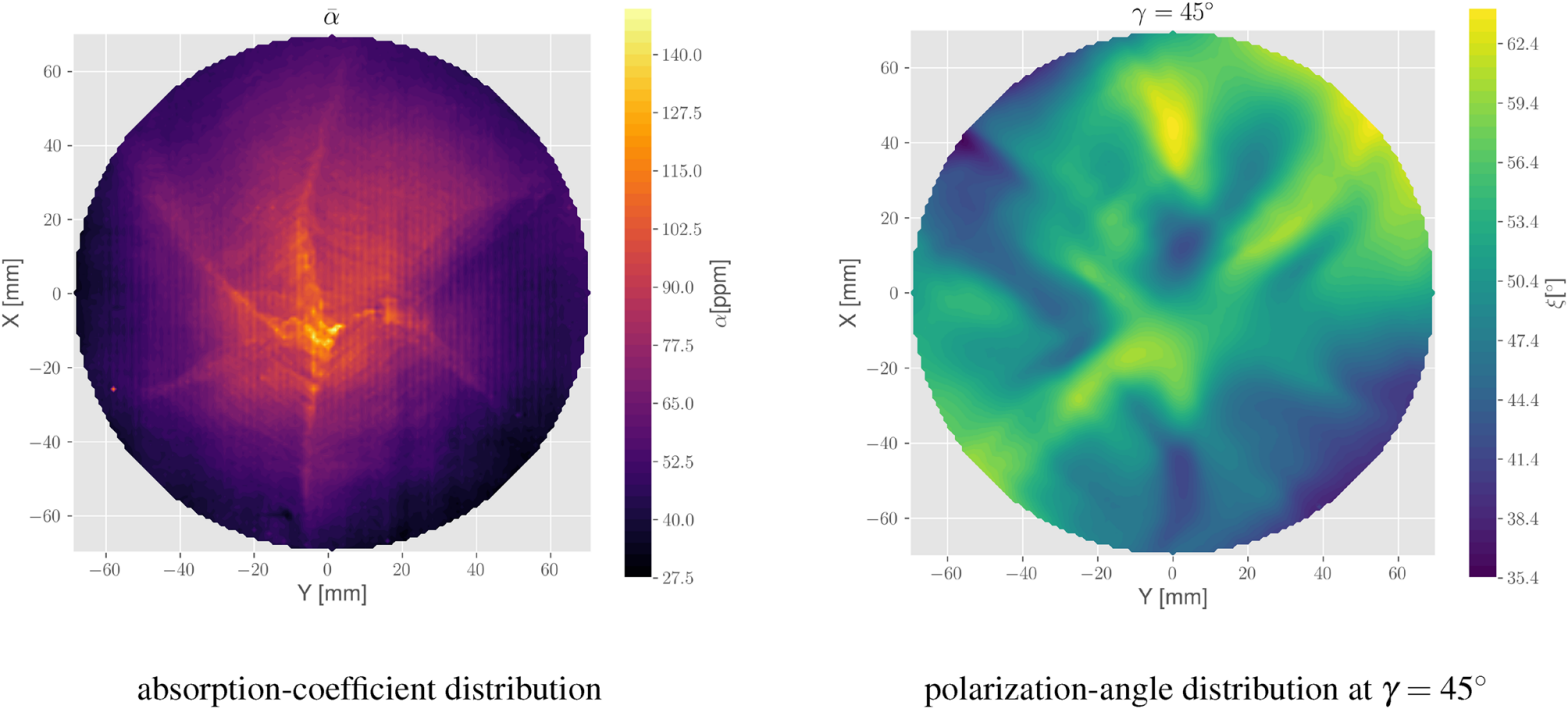
Left: an overview of the mean absorption coefficient distribution for the studied sample. It is the identical sample as presented already by Marchiò et al.^11^, but for the purpose of this paper, we have remeasured it and took an average of seven XY sections at different depths along its thickness. Right: map showing the birefringence effect in terms of the polarization angle $\xi$.

Before performing the birefringence measurement, we characterized the sample in terms of the absorption coefficient distribution using the PCI method^11^. As the PCI setup and the birefringence setup are built on the same optical table and share the laser source and the same sample positioning system, the two maps can be conveniently superimposed in order to evidence any correlation between the linear absorption and the birefringence maps.

In Fig. 3 the absorption coefficient distribution of the sample is presented. We limited map-taking to a region around the center axis of each sample, keeping usually a margin of $\gtrsim 1/3$ of a sample diameter to the outer edges. Birefringence maps provide information that is necessarily averaged along the whole sample thickness, while the PCI absorption setup performs 3D spatially resolved measurements. Thus, in order to perform a comparison, we computed an average of 7 absorption XY sections obtained at different thicknesses along the sample. Obviously, the comparison is here meant to remain at the qualitative level, but nevertheless, it should be noted that the main structures in absorption appear to be relatively invariant along Z. Thus, a direct comparison with birefringence maps is indeed reasonable.

The absorption map shows a star-like pattern with a clear absorption peak in the center. The rms variation of the absorption coefficient over the entire map is $\sim 61.56\pm 17.11$ ppm/cm. Fringes are visible with orientations according to each sub-segment. The distance between the fringes appears to be of the order of a few millimeters. The star-like pattern and the fringes are probably remnants from the manufacturing process (“growth striations”)^8,11^. Particularly the pattern reflects the symmetries of the crystal structure of Sapphire. The map in Fig. 3 has been taken with a lateral step size of $0.5 {{{\hbox {mm}}}}^{2}$ and $1 {{{\hbox {mm}}}}^{2}$, respectively.

### Birefringence maps

**Figure 4:**
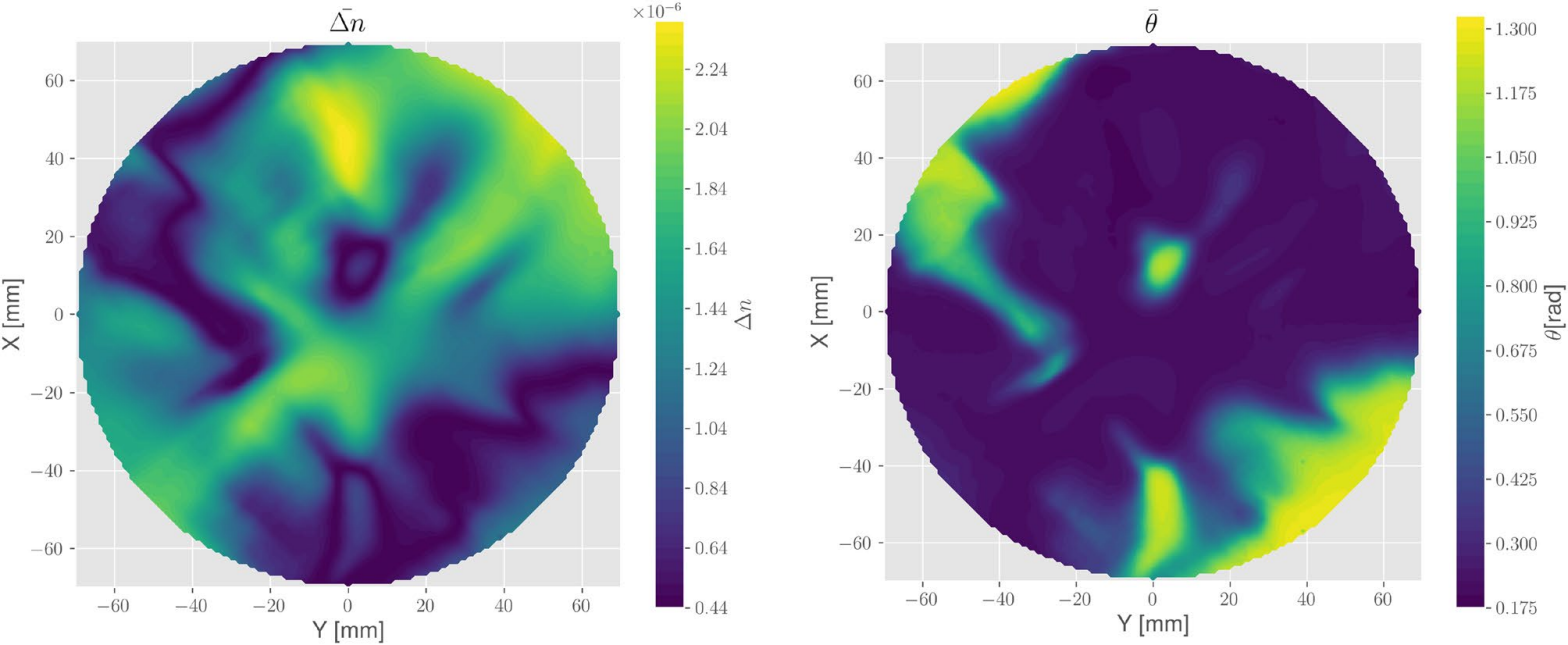
Mean distribution of both birefringence $\Delta n$ and $\theta$-angle, calculated from the six input-polarization combinations which led to no miscalculations.

In Fig. 3 on the right, a map of the “polarization angle” $\xi$ from our sample is presented which we defined from the ratio of the normalized power of the electric fields $P_s$ and $P_p$ from the propagated light (see also Fig. 2) as:

7 $$\begin{aligned} \xi = \arctan \left( \sqrt{\frac{P_s}{P_p}}\right) , \end{aligned}$$

with $P_s$ and $P_p$ being the measured (normalized) power of the s and p polarized components of the transmitted beam. The input polarization was $\gamma =45$°, while the orientation of the sample remains the same as for the absorption measurements. When inspecting the absorption and polarization-angle distributions, we can spot a correlation between their main features, particularly the star-like pattern which we clearly observe in absorption, while fringes cannot be observed in the polarization-angle distribution.

The given distribution in terms of $\xi$ is direct evidence that birefringence inhomogeneities introduce a measurable distortion of the optical beam and shows the basic problem of using Sapphire substrates in gravitational wave detectors like KAGRA. To better investigate the birefringence distribution in our sample, we have taken several maps as the one shown in Fig. 3 with different input polarization $\gamma$ and extract the information of $\Delta n$ and $\theta$ according to Eqs. (4) to (6).

$\gamma$ has been varied from $0^\circ$ (S-polarized) to $75^\circ$ (in steps of $15^\circ$). From the resulting maps, we calculated the distribution of $\Delta n$ and $\theta$ for each combination of input-polarization angles, resulting in 15 independent pairs of $\Delta n$ and $\theta$ distributions. In theory, the choice of $\gamma$ does not affect the result of the measurement. However, as worked out in the section “Experimental setup”, the uncertainty of the measured power $P_s$ and $P_p$ is of the order $10^{-4}$ which becomes crucial whenever either polarization state in the transmitted beam is very small. Hence, we had to exclude those combinations where the uncertainty in one of the maps leads to areas of miscalculation in either $\Delta n$ or $\theta$. The remaining 6 combinations for maps taken at 30, 45, 60, and $75^\circ$ have been used for further analysis. We can also compute our uncertainty in $\Delta n$ to be $2 \times {10}^{-9}$ from the uncertainty of the measured power together with uncertainty in our input polarization $\gamma$.

Those maps should theoretically coincide. Variations in the obtained values can be thus taken as an estimation of the method’s reproducibility. The average maps for both parameters have been calculated from these quantities and are plotted in Fig. 4. The structures that appeared in the $\xi$-angle map are defined by the $\Delta n$ distribution, which is also a measure of the magnitude of internal stress. It is indeed possible to give an estimation of that stress with the aid of the photo-elastic tensor^17,18^. However, due to the missing resolution in Z, only a projected effective stress parameter could be calculated from $\Delta n$, which resulted in a maximum value of $\sim 0.54 \,{{\hbox {MPa}}}$. As can be seen, a meandering pattern defines the minimum in $\Delta n$ which marks at the same time a border between $\theta > 0$ and $\theta < 0$. In fact, $\theta$ would further increase above values of $\pi /4$ along that border, but due to the arctangent in Eq. (6), it is wrapped in intervals of length $\pi /2$. As we want to avoid the discontinuities that follow from the wrapping, we unfolded the map to show the continual distribution of $\theta$ which is therefore given in the interval $[0, \pi /2]$. We also see a relatively strong gradient along that border with $\theta$ reaching values of $\sim 1.4$. In other areas, $\theta$ is relatively constant between 0.1 and 0.25 (all values for $\theta$ are given in radians)

**Figure 5:**
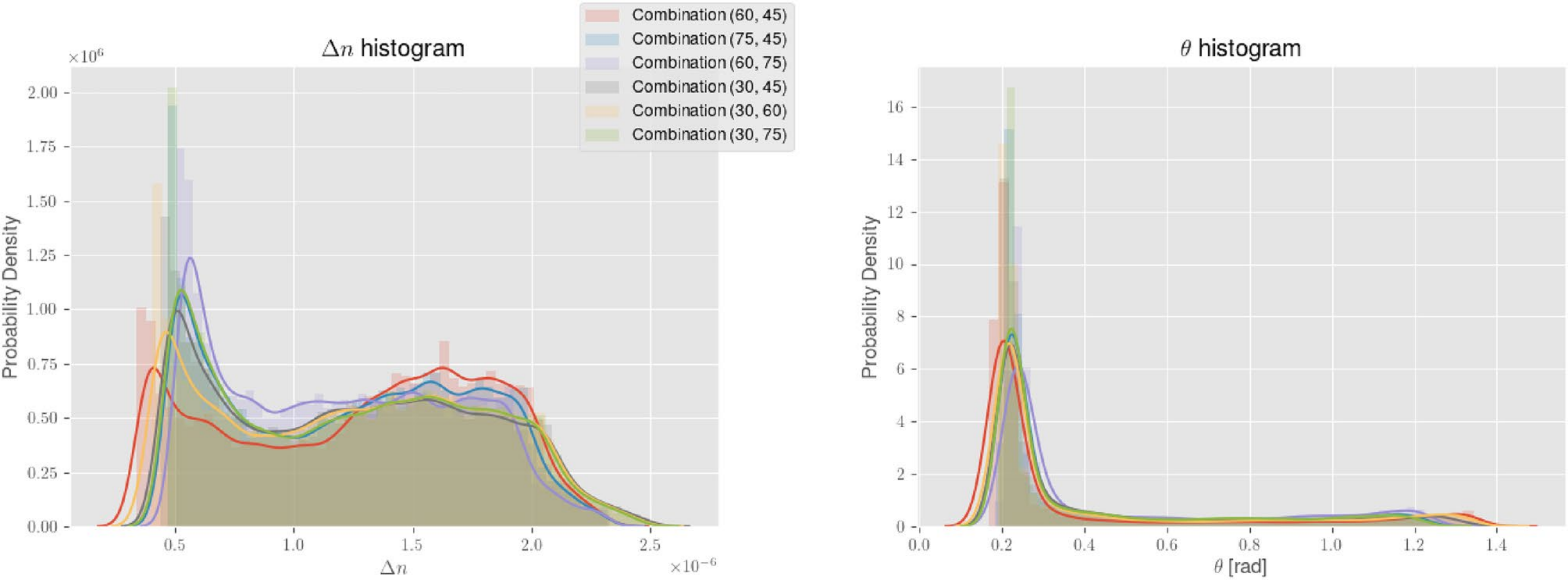
Histograms of both $\Delta n$ and $\theta$ parameter distributions for all 6 combinations of input polarization angles that resulted in no miscalculations. The histograms are plotted in terms of their probability density together with an estimated probability density function.

At this point, we also mention some statistics regarding $\Delta n$ and $\theta$ from each combination. In Fig. 5, the probability density histograms of these parameters for all 6 combinations of input polarization angles are presented. As mentioned above, the maps should be physically identical, independent of $\gamma$, and so should also the histograms. Despite minor discrepancies, this seems to be the case for both $\Delta n$ and $\theta$. The minimum of $\Delta n$ lies at $3\sim 5\times 10^{-7}$ where we recognize a peak at this position for all distributions. Taking into account the noise floor of the lock-ins, the measurements should enable us to resolve the birefringence down to $\sim 10^{-7}$. Therefore our result may indicate that we have just reached the measurable range in many areas of the maps. Another reason could be attributed to a small miscut of the c-axis with respect to the major surfaces normal. A c-axis misalignment for example of the order $0.2^\circ$ would have the same effect as a birefringence of $\sim 1.5 \times 10^{-7}$ for small $\theta$ values. This, however, should be excluded if the sample is in spec.

The histogram for $\theta$ reflects our main observation from Fig. 4. We see a dominating peak at $0.15\sim 0.3$ and a continuity towards larger values at low-density levels, which eventually passes $\pi /4$ toward a maximum of $\sim 1.4$.

## Discussion

We have shown the effect of birefringence in large Sapphire substrates on the polarization of a transmitted laser beam. From the information we draw from the direct measurements of $V_p$ and $V_s$ as well as the other parameter defining our system, we could conclude on the birefringence orientation ($\theta$) and its strength ($\Delta n$).

**Figure 6:**
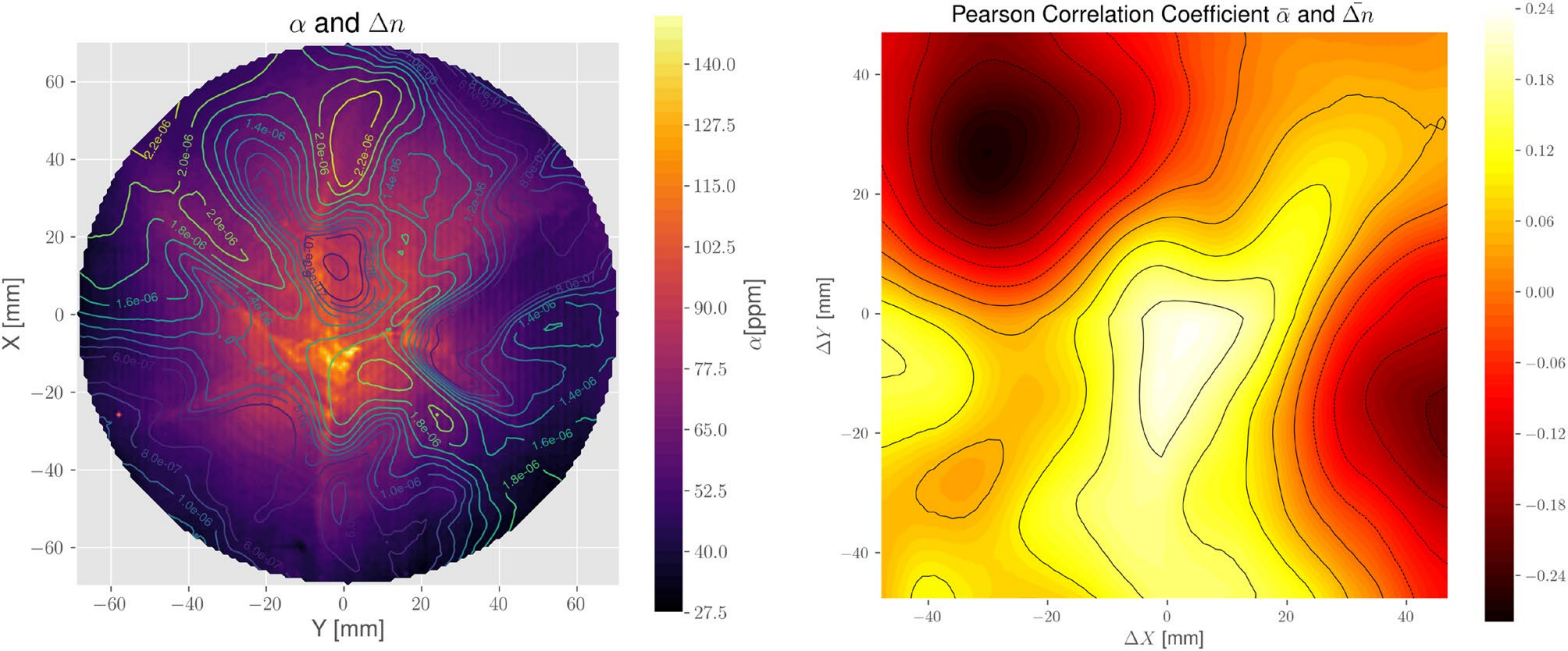
Left: overlay plot of the mean-absorption map shown in Fig. 3 and the mean $\Delta n$ distribution. The latter is given as a contour plot with the respective values given inside the figure. Right: Pearson correlation coefficient in two dimensions between the absorption and the birefringence distribution, calculated from a quadratic cut from both maps with a maximum number of valid values.

The sample’s birefringence appears to be related to the absorption-coefficient distribution, which in turn may have its origin in the manufacturing process^8,11,19^ (this has been pointed out also by previous research using transmitted wavefront error maps^20^). In Fig. 6, an overlay plot of both the absorption coefficient as given in Fig. 3 and $\Delta n$ as given in Fig. 4 is presented. Both are functions of the spatial parameters *X* and *Y*, and can thus be analyzed in terms of their Pearson correlation coefficient (that is the zero-normalized cross-correlation), which gives a maximum of $\sim 0.24$ at $\Delta X = \Delta Y = 0$, hinting indeed to a correlation (see Fig. 6). Moreover, the shape of the correlation map clearly shows that the maximum resides in the center, surrounded by local minima. Given the specific shape of a six-fold star-like pattern in the absorption map, that particular correlation indicates that the same shape must exist also in the birefringence map. A differently shaped map would result in a completely different correlation pattern (see also the supplementary material to this paper). The elongated elevation of the correlation in Fig. 6 from bottom-left to top-right on the other side is due to the differences and specific structures in the birefringence map compared to that of the absorption.

Please note that we have cut out a maximum quadratic area from both maps to calculate the correlation with valid values only. This minimizes the bias which would otherwise overstate the correlation. In the case of the most prominent features in $\Delta n$ we see that they lie either in between or close to the radial absorption lines forming the aforementioned star-like structure. Exceptions from this correlation are only present at the edges of the measured area. It has been pointed out already that the star-like structure visible in the absorption map actually marks the edges of growth planes^11^ which are known to attract impurities such as transition elements or vacancies. Point defects, on the other side, may act as a source of compositional stress. From the results presented here, we see increased birefringence also in regions where no absorption centers are visible (e.g., in the bottom area). This may be a hint for the existence of impurities in those regions that do not act as absorption centers at $\lambda = 1064 \, {{\hbox {nm}}}$ but at different wavelengths or remaining stress induced by thermal gradients from manufacturing. Unfortunately, the birefringence data are not sufficient to fully reconstruct the whole stress tensor. At the moment it appears difficult to relate quantitatively the stress field with the impurity distribution and it would be a task for future investigation. Also, having a model on the connection between birefringence and absorption would be very helpful for further understanding of our characterization. This, however, is beyond the scope of this paper.

We could show that there is apparently one preferred direction of birefringence as $\theta$ is accumulating at values $\sim 0.25 \, {{\hbox {rad}}}$ (or $\sim 14^\circ$). The reason for this preferred direction, however, is yet unknown. At this point, it should be mentioned that due to the system’s limitations, we could perform the measurements only at room temperature, whereas KAGRA operates the mirrors at $20\, {{\hbox {K}}}$. While there is most likely an effect on stress, we cannot quantify it here. However, we may note that if $\Delta n$ shows only low variations at 300 K, we can expect a similar situation also at lower temperatures. That is due to the fact that the actual reason we see for the measured birefringence (internal stress due to impurities and dislocation networks) remains even at low temperatures, while the elasto-optical tensor of the sapphire crystal is most probably only weakly depending on temperature.

If we follow the argument that local stress due to vacancies, impurities, or other imperfections are the origin of crystal-structure (and hence birefringence) inhomogeneities, then sample annealing could be a possible way to reduce it to a certain extent. In fact, oxygen vacancies, diffusive impurities, and defect clusters may be redistributed, leading to a more homogeneous defect concentration and thus smoothing out the stress fields^8^. On the other hand, impurities in oxides are optically active at wavelengths in the ultra-violet and visible range^21,22^ and in most cases, we can clearly identify them in UV-VIS spectra from Sapphire samples smaller than the one presented in this paper as a relatively strong and broad absorption band at around $250 \sim 260\,{{\hbox {nm}}}$ wavelength, which is a clear indication of F+ type color centers^23,24^. At longer wavelengths, we were able to identify several other absorption bands (especially at around 350, 400, and $450 \, {{\hbox {nm}}}$). In particular a band at $400 \, {{\hbox {nm}}}$ is probably due to Cr$^{3+}$ ions^23^. Transition-metal impurities are substitutional defects that are strongly bonded within the oxygen cage which constitute the Sapphire structure. Those defects are difficult to be removed by annealing. However thermal treatments can be used at least to modify their oxidation state^24^, which eventually will remove at least their contribution to absorption. Unfortunately, our device is not sensitive enough to investigate the (cumulative) birefringence effect in smaller samples which are otherwise measurable by spectrometers (and usually optically more isotropic), so a direct comparison between birefringence and spectroscopy could not be established yet.

## Conclusion

We developed a system that is able to perform polarization measurements on large-size sapphire samples to be used as test masses for gravitational wave detectors. Using this system we were able to obtain position-resolved birefringence data. The setup is integrated within the PCI system at the TAMA laboratory in NAOJ (Tokyo) which results in a unique compilation of having both absorption and birefringence distributions of a large sample without changing its orientation.

We could show for the KAGRA-sized test sample that there is a correlation between the distribution of the absorption coefficient and the birefringence. Due to the results, we can say that the compositional absorption inhomogeneities are an important factor for increased internal stress and thus the birefringence structures which we observed. For the first time, we were able to analyze these structures on Sapphire samples having sizes above 200  mm diameter and thicknesses of 150 mm. Although the actual effect of the birefringence inhomogeneities may be negligible for thinner samples, our work proves the necessity in gravitational-wave detector science to be able to measure thick samples since the cumulative effect increases linearly with the thickness and may be underestimated by concentrating on small samples only. At the same time, our work is also important for future manufacturing of Sapphire samples as it provides information for manufacturers to analyze their product using non-standard methods not available to the crystal manufacturers.

### Supplementary Information

Supplementary Figure S1.

## Data Availability

The datasets used and/or analyzed during the current study are available from the corresponding author on reasonable request.
