# Supplementary material for: Correlation between birefringence and absorption mapping in large-size Sapphire substrates for gravitational-wave interferometry
Source: Sci Rep. 2023 Dec 4;13:21393. doi: 10.1038/s41598-023-45928-0 (PMC10696018; doi:10.1038/s41598-023-45928-0)
Supplement: Supplementary file 1 — Supplementary Figure S1. [file 41598_2023_45928_MOESM1_ESM.pdf]

In order to size the reliability of our results reported Fig. 6 from the standpoint of indicating a meaningful relationship between absorption and birefringence maps we performed some additional numerical tests.

We numerically generated a noisy six-fold star-pattern and computed the correlation of such an image with a set of test patterns in the shape of noisy n-fold stars. In all cases the added noise is normally distributed with a variance of 1 (the amplitude of the pattern is also 1, hence the noise is relatively strong).

As can be seen, the only case where Pearson's correlation map shows a distinct maximum in the center is for the case of two six-fold patterns with the same size and position (Fig. S1 (d). The maximum correlation in the center of the map is as high as 0.36, which is almost two times higher than any other combination explored.

We also notice that this maximum is surrounded by the minima of the correlation-map.

Taking our result from the absorption and birefringence measurements, we recognize a very similar behavior in the correlation. We too see a distinct maximum in the center and areas of a minimized correlation surrounding it.

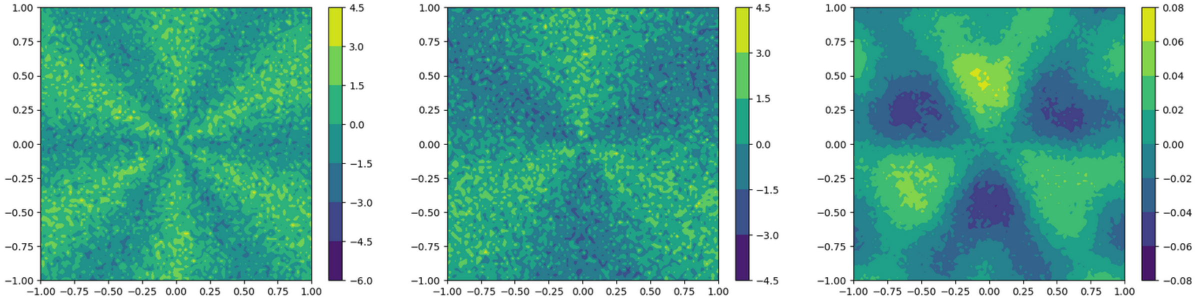

(a)

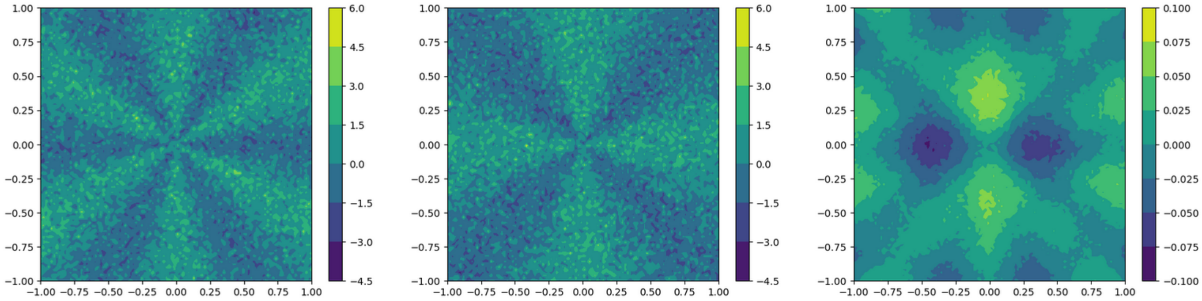

(b)

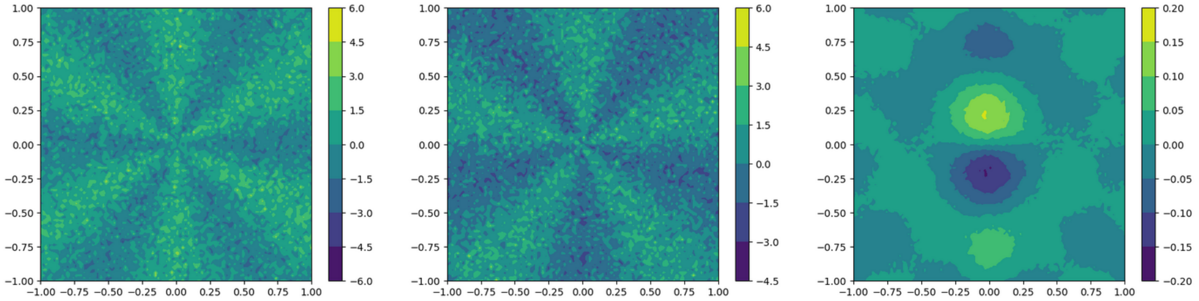

(c)

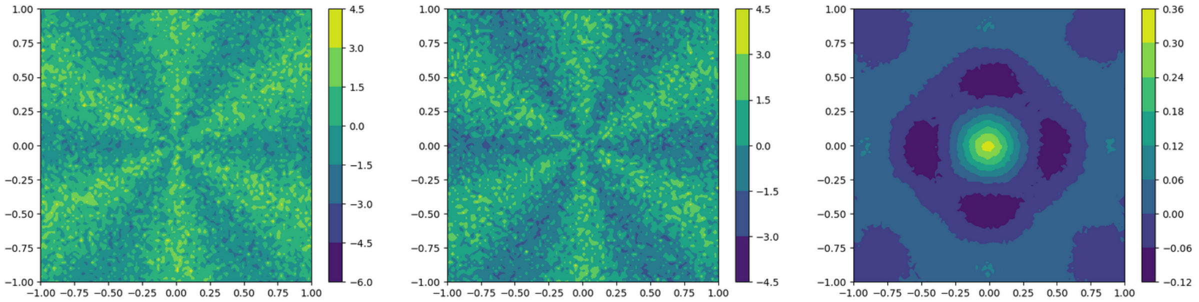

(d)

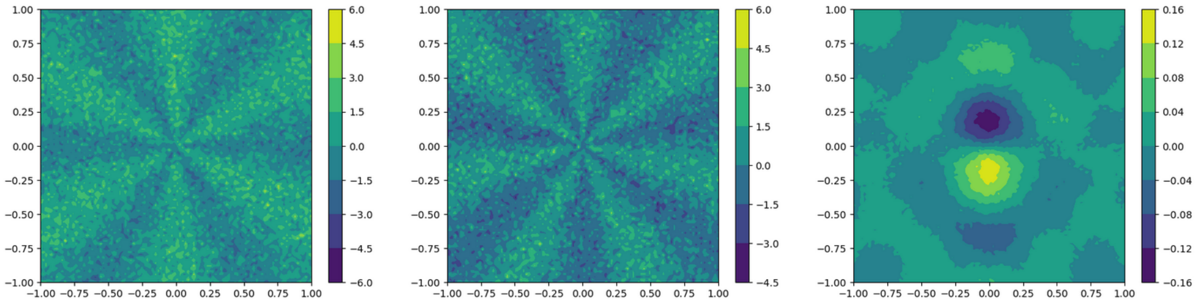

(e)

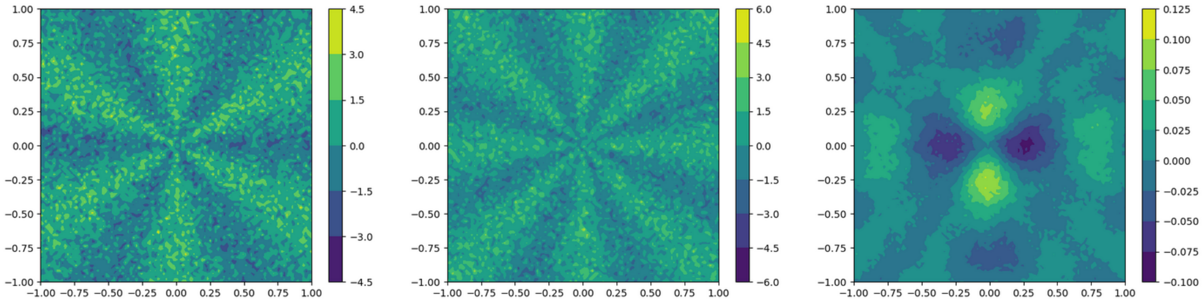

(f)

Given the very specific shape of the six-fold star-like pattern in the absorption map, our numerical studies prove that a similar shape must exist also in the birefringence map. Obviously, the measured birefringence and absorption maps have a more complex structure, which is the reason why additional features can be observed in the correlation map of Fig. 6 (such as the broad “cloud” extending from the bottom-left to the top-right of the map. However, the presence of the single maximum in the center of the correlation map with a value as high as 0.24 testifies the similarity between the absorption and the birefringence maps beyond a mere statistical fluctuation, as proven by our simulations.
